# ENDOSCOPIC CHARACTERISTICS OF PATIENTS WITH COMPLETE PATHOLOGICAL RESPONSE AFTER NEOADJUVANT CHEMOTHERAPY FOR GASTRIC AND ESOPHAGOGASTRIC JUNCTION ADENOCARCINOMAS

**DOI:** 10.1590/0102-672020210002e1616

**Published:** 2021-12-17

**Authors:** Juliana Silveira Lima de CASTRO, Adriane Graicer PELOSOF, João Guilherme Guerra de ANDRADE-CABRAL, Alvaro Moura SERAPHIM, Eloy TAGLIERI, Felipe Jose Fernandez COIMBRA, Claudia ZITRON

**Affiliations:** 1 A.C. Camargo Cancer Center, Endoscopy Unit, São Paulo, SP, Brazil; 2 A.C. Camargo Cancer Center, Gastrointestinal Surgical Oncology Unit, São Paulo, SP, Brazil

**Keywords:** Neoadjuvant therapy, Stomach neoplasms, Treatment outcome, Endoscopy, digestive system, Neoplasm staging, Terapia neoadjuvante, Neoplasias gástricas, Resultado do tratamento, Endoscopia do sistema digestório, Estadiamento de neoplasias

## Abstract

**Background:**

Gastric and esophagogastric junction adenocarcinoma are responsible for approximately 13.5% of cancer-related deaths. Given the fact that these tumors are not typically detected until they are already in the advanced stages, neoadjuvancy plays a fundamental role in improving long-term survival. Identification of those with complete pathological response (pCR) after neoadjuvant chemotherapy (NAC) is a major challenge, with effects on organ preservation, extent of resection, and additional surgery. There is little or no information in the literature about which endoscopic signs should be evaluated after NAC, or even when such re-evaluation should occur.

**Aim:**

To describe the endoscopic aspects of patients with gastric and esophagogastric junction adenocarcinomas who underwent NAC and achieved pCR, and to determine the accuracy of esophagogastroduodenoscopy (EGD) in predicting the pCR.

**Methods:**

A survey was conducted of the medical records of patients with these tumors who were submitted to gastrectomy after NAC, with anatomopathological result of pCR.

**Results:**

Twenty-nine patients were identified who achieved pCR after NAC within the study period. Endoscopic responses were used to classify patients into two groups: G1-endoscopic findings consistent with pCR and G2-endoscopic findings not consistent with pCR. Endoscopic evaluation in G1 was present in an equal percentage (47.4%; p=0.28) in Borrmann classification II and III. In this group, the predominance was in the gastric body (57.9%; p=0.14), intestinal subtype with 42.1% (p=0.75), undifferentiated degree, 62.5% (p=0.78), Herb+ in 73.3% (p=0.68). The most significant finding, however, was that the time interval between NAC and EGD was longer for G1 than G2 (24.4 vs. 10.2 days, p=0.008).

**Conclusion:**

EGD after NAC seems to be a useful tool for predicting pCR, and it may be possible to use it to create a reliable response classification. In addition, the time interval between NAC and EGD appears to significantly influence the predictive power of endoscopy for pCR.

## RESUMO

**Racional:**

O adenocarcinoma gástrico e da junção esofagogástrica é responsável por aproximadamente 13,5% das mortes relacionadas ao câncer. Dado que esses tumores não são normalmente detectados até que já estejam em estágios avançados, a neoadjuvância desempenha um papel fundamental na melhoria da sobrevida em longo prazo. A identificação daqueles com resposta patológica completa (pCR) após a quimioterapia neoadjuvante (NAC) é um grande desafio, com efeitos na preservação do órgão, extensão da ressecção e cirurgia adicional. Há pouca ou nenhuma informação na literatura sobre quais sinais endoscópicos devem ser avaliados após a NAC, ou mesmo quando essa reavaliação deve ocorrer.

**Objetivo:**

Descrever os aspectos endoscópicos de pacientes com adenocarcinoma gástrico e da junção esofagogástrica que foram submetidos à quimioterapia neoadjuvante e alcançaram pCR, e determinar a acurácia da esofagogastroduodenoscopia (EGD) em predizer a pCR.

**Métodos:**

Foram revisados os prontuários de pacientes submetidos à gastrectomia subtotal e total após NAC, com resultado anatomopatológico de pCR.

**Resultados:**

Vinte e nove pacientes que alcançaram pCR após NAC foram identificados no período estudado. As respostas endoscópicas foram usadas para classificar os pacientes em dois grupos: G1- achados endoscópicos consistentes com pCR, G2 - achados endoscópicos não consistentes com pCR. A avaliação endoscópica no G1 esteve presente em igual percentual (47,4%; p=0,28) na classificação de Borrmann II e III. Nesse grupo, a predominância foi no corpo gástrico (57,9%; p=0,14), subtipo intestinal com 42,1% (p=0,75), grau indiferenciado, 62,5% (p=0,78), Herb+ em 73,3% (p=0,68). O achado mais significativo, no entanto, foi que o intervalo de tempo entre NAC e EGD foi maior para G1 do que G2 (24,4 vs. 10,2 dias, p=0,008).

**Conclusão:**

A EGD após NAC, nessa pesquisa, sugeriu ser método útil para prever pCR, mediante uma classificação de resposta confiável. Além disso, o intervalo de tempo entre NAC e EGD parece influenciar significativamente a sua capacidade preditiva de diagnosticar a pCR.

## INTRODUCTION

Despite a reduction in the incidence of gastric adenocarcinoma (GC) in the last decade, it remains the third most common cause of cancer-related death in the world, with an estimated 783,000 deaths per year^5^.The incidence of adenocarcinoma of the esophagogastric junction (GEJ) has increased markedly in Western countries in recent years^5^ ^,^ ^4^. Population analyses in the United States have reported an almost 2.5-fold increase in incidence since the 1970s^14^. In Brazil, GC is the 5^th^ most common cancer overall (3^th^ among men and 5^th^ among women)^15^. For each year from 2020 to 2022 it has been projected that there will be 13,360 new cases in men and 7,870 new cases in women per hundred thousand people^15^. For esophageal adenocarcinoma in Brazil, there are expected to be 8,690 new cases in men and 2,700 new cases in women per hundred thousand^15^. GC development can be induced by the interactions of multiple genetic and environmental factors in complex ways^23^.

Currently, the recommended therapeutic approach for locally advanced tumors of the stomach and GEJ is perioperative chemotherapy^3^ ^,^ ^7^. Several studies have demonstrated this strategy to yield increased rates of complete resection, downstaging, overall survival, and progression-free survival^3^ ^,^ ^8^. It has also been noted that some tumors exhibit better responsiveness than others^7^ ^,^ ^8^. Therefore, diagnostic methods that can predict a complete pathological response (pCR) have important clinical implications^7^ ^,^ ^22^. Several combined chemotherapy regimens have shown good efficacy for GC and non-resectable GEJ allowing a potential curative gastrectomy^22^. Among these regimens, the most used today is the FLOT scheme, composed of fluorouracil, leucovorin, oxaliplatin, and docetaxel^2^. In patients with locally advanced lesion above clinical stage tumor (cT) 2 or compromised lymph nodes, neoadjuvant chemotherapy (NAC) can increase the likelihood of curative surgery, with complete tumor response rates around 10% and increased rates of both progression-free and overall survival^9^ ^,^ ^21^. In addition, NAC can offer treatment options for patients for whom surgery is risky, such as those with more advanced disease progression^4^. The identification of patients with pCR after NAC could, in the future, become a tool to select those who really benefit from adjuvant chemotherapy and perhaps even in the suppression of surgery to patients at high risk for the procedure, becoming a useful tool in the decision multidisciplinary therapy^4^. In general, the morbidity rate of radical gastrectomies is around 33.5% and mortality between 0.6% to 4.7%^1^ ^1^ ^,1^ ^6^. According to a 2015 study the overall survival of stage III/IV patients who underwent NAC and who obtained pCR was similar to those with stage I/II who did not receive NAC^6^. Recently published data demonstrated that pathological staging was better than conventional staging at predicting responsiveness to and survival after neoadjuvancy^7^. Other studies have also indicated that location in GEJ and TNM are associated with a worse prognosis^8^. Preoperative endoscopic evaluation of patients undergoing NAC is recommended in many services, but as of the writing of this paper there has been no published description of endoscopic findings in these patients and the ideal time interval between NAC and surgery remains unclear. 

Therefore, in the present study we investigated the following questions: 1) What are the endoscopic features that support detection of pCR following NAC? 2) What is the diagnostic accuracy and sensitivity of esophagogastroduodenoscopy (EGD) in the assessment of pCR following NAC? and 3) What time interval between NAC and EGD supports optimal response prediction?

## METHODS

This is a retrospective study in a single center specializing in cancer treatment. The medical records were revised of patients with GC and GEJ type adenocarcinoma who were submitted to gastrectomy after NAC with an anatomopathological result of pCR. 

From October 2010 to September 2018, we identified 31 patients aged >18 years who underwent total or subtotal gastrectomy after NAC for the treatment of CG and GEJ and who exhibited pCR. All patients were treated at A.C. Camargo Cancer Center, São Paulo, SP, Brazil. Clinical stages of patients ranged from cT2-cT4. Were excluded two patients that missed the examinations. The study was approved by the ethics and research committee of A.C. Camargo Cancer Center, under number: 2892/20.

### Study design

All EGDs were performed by two senior endoscopists using 150 and 180 videoendoscopies (Olympus Medical System Corporation, Hachioji-shi, Tokyo, Japan).

#### Esophagogastroduodenoscopy 1 (EGD1)

Patients underwent EGD with biopsy and anatomopathological studies that confirmed GC or GEJ adenocarcinomas. At this time the macroscopic aspect of the lesion was classified according to Borrmann classification (BC) as: BC-I, well-defined polypoid or vegetating lesion; BC-II, ulcerated lesion, well-delimited with clear edges; BC-III, ulcerated lesion, infiltrative in part or all of its borders; BC-IV, diffusely infiltrative lesion, with no limit between the tumor and the normal mucosa. 

After the histopathological diagnosis patients were staged using a computed tomography (CT) scan of the chest, abdomen, and/or pelvis and fluoro-2-deoxyglucose positron emission tomography/computed tomography (FDG-PET/CT). 

Patients underwent NAC with a scheme based on fluoracil and platinum, with either 8 or 12 cycles, half of the sessions being performed before surgery and the remainder in the postoperative period. 

#### Esophagogastroduodenoscopy 2 (EGD2)

Within 30 days of the end of preoperative NAC, all patients again underwent EGD and were reclassified according to the macroscopic aspect of the lesion. After NAC, patients underwent partial or total gastrectomy with D2 lymphadenectomy and surgical specimens were processed according to standard procedures. 

The histopathological studies of surgical specimens were performed using the World Health Organization's classification scheme for neoplasms of the digestive system as well as Lauren's classification, with the following characteristics recorded: subtype, Lauren type, depth of invasion in the wall, lymph node status, vascular and neural infiltration^1^ ^9^. 

All surgical specimens were analyzed by two independent pathologists using the tumor regression score as recommended by the National Comprehensive Cancer Network^1^. According to this scale, a score of zero indicates complete response and comprises no viable cancer cells, including in lymph nodes. All of the 29 patients included in this study showed scores of zero and complete anatomopathological responses.

### Objectives and definitions

The primary objectives of this study were to describe the endoscopic aspects of patients with gastric or GEJ adenocarcinomas submitted to NAC who exhibited pCR, and to determine the accuracy of EGD in predicting this response in these patients. 

**FIGURE1:**
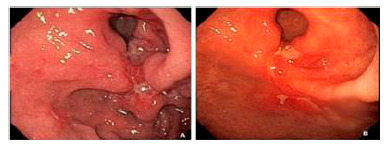
Group 1: A) Esophagogastroduodenoscopy pre-neoadjuvant chemotherapy showing Borrmann classification-III ulcerated lesion located in cardia; B) esophagogastroduodenoscopy post- neoadjuvant chemotherapy showing scar.

**FIGURE 2:**
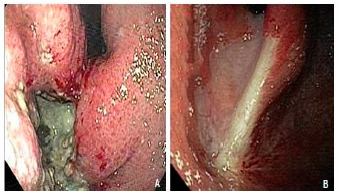
Group 2: A) Esophagogastroduodenoscopy pre-neoadjuvant chemotherapy showing Borrmann classification-III ulcerated lesion located at antrum; B) esophagogastroduodenoscopy post-neoadjuvant chemotherapy showing active lesion

A complete endoscopic response (eCR) was determined by the presence of an endoscopic scar (reddish or whitish) without active lesions after NAC (Figure 1A/B). Patients who met this criterion were included in group 1 (G1, n=19). Patients who exhibited active lesions (ulcers) after NAC were included in group 2 (G2, n=10; Figure 2A/B). 

Secondary objectives of this study were to evaluate whether factors such as gender, age, tumor location, BC, cT, lymph node status, histopathological subtype, degree of differentiation, Herb2 marker status, and time interval between NAC and EGD2 may influence the sensitivity of EGD to predict pCR. 

### Statistical analysis

All statistical analyses were performed using IBM SPSS software version 25. Statistical significance was set as p<0.05. Absolute (n) and relative (%) frequency distributions were evaluated for qualitative variables, and main summary measures (mean, standard deviation, median, minimum, maximum) for quantitative variables. Chi-square and Fisher's exact tests were used to determine the association between qualitative variables and the presence of scarring. The Mann-Whitney non-parametric test was used to associate quantitative variables with the presence of scar. If an association was found between scar and any variable, logistic regression was used to calculate the odds ratio. For evaluation of the scar in relation to pCR, the main diagnostic measures (sensitivity, specificity and accuracy) were calculated.

## RESULTS

Characteristics of patients and tumors are summarized in Table 1. Although there were more males enrolled than females, genders were distributed similarly across groups (p=0.22).

The G1 group compared to G2 group, more patients presented with GC (73.7%, p=0.67) cT 3 (68.4%, p=0.72), and absent lymph node status (68.4%, p=0.59). After EGD, the lesions were classified in equal proportions as BC-II and BC-III (47.4%, p=0.28), more in the gastric body (57.9%, p=0.14) and the histopathological study showed that, the intestinal subtype was present in 42.1% (p: 0.75), undifferentiated degree, 62.5% (p=0.78), Herb + in 73.3% (p=0.68). 

The time interval between the last NAC cycle and the realization of the reevaluation by EGD was higher in G1 compared to G2. In G1 this average was 24.4 days [minimum: 5 days; maximum: 61 days, standard deviation (SD): 16] and in G2 the mean was 10.2 (minimum: 2, maximum: 5, SD: 10.4), (p: 0.008), odds ratio (OR) 1.12 and [confidence interval (CI): 1.0-1.2]. mean 24.4 days (5- 61), standard deviation (SD): 16] and 10.2 (minimum: 2, maximum: 5, SD: 10.4) respectively (p: 0.008), odds ratio (OR) 1.12 and [confidence interval (CI): 1.0-1.2]. 

The ideal time calculated for performing EGD after NAC was 8.5 days. At this time, a sensitivity of 93% and specificity was reached: 66% (CI: 0.6-1.0). EGD after NAC showed accuracy in predicting endoscopic complete response (eCR) and sensitivity, G1, in 65.5% of the analyzed cases. All cases underwent surgical treatment with partial gastrectomy, total gastrectomy with or without distal esophagectomy associated with D2 lymphadenectomy, with a finding of pCR in the surgical specimen in an average of 43.8 days in both groups, with the average in G1 being 52, and in G2 39 days after endoscopic control.

**TABLE 1:** Characteristics of patients and tumors

Variable	G1		G2		
	n	%	n	%
Age (years)	58.3	-	65.1	-
Gender				
Male	14	73,6	8	80
Female	5	26,3	2	20
Siewert Classification				
II	5	26.3	4	40
III	14	73.7	6	60
Localization				
Cardia	5	26.3	4	40
Body	11	57.9	2	20
Antrum	3	15.7	4	40
Grade				
Poorly differentiated	10	52.6	7	70
Moderately differentiated	5	26.3	2	20
Well differentiated	1	5.2	0	0
Unknown	3	15.7	1	10
Signet ring cell histology				
Absent	9	47.3	5	50
Present	10	52.6	5	50
cT category				
1	0	0	0	0
2	3	15.7	3	30
3	13	68.4	6	60
4	3	15.7	1	10
cN status				
Negative	13	68.4	9	90
Positive	6	31.5	1	10
cM category	0	0	0	0
Endoscopic findings before chemotherapy				
BC-I	0	0	0	0
BC-II	9	47.3	2	20
BC-III	9	47.3	8	80
BC-IV	1	5.2	0	0
cHerb				
Positive	2	10.5	3	30
Negative	11	57.8	5	50
Unknown	2	10.5	1	10
Histological type				
Diffuse	7	36.8	4	40
Intestinal	8	42.1	2	20
Mixed	4	21	2	20
Type of resection				
Total gastrectomy	10	52.6	2	20
Subtotal gastrectomy	9	47.3	8	80
Lymph node dissection				
D1	0	0	0	0
D1+/D2	19	100	10	100
Endoscopic findings during or after chemotherapy				
Scar	19	100	0	0
Lesion	0	0	10	100

cT=clinical stage tumor; cN= clinical stage lymph nodes; cM=clinical stage metastasis; BC= Borrmann classification

## DISCUSSION

GC is one of the most common neoplasms in the world, with high rates of incidence and mortality^20^. The most common location is the gastric antrum although the incidence of GEJ tumors is gradually increasing^1^ ^3^. GEJ tumors are very prevalent worldwide and are among the most aggressive tumors of the digestive tract^5^. Furthermore, in most western countries they are not diagnosed until the more advanced stages, when isolated surgical treatment is less effective^6^. The low rate of early gastric cancer is related to the lack of specific symptoms^1^ ^9^. Advanced tumors exhibit considerable metastatic potential and generally worse prognosis, indicating a need for combined systemic and local treatments to reduce the risk of tumor recurrence^12^. 

Complementary treatments associated to radical surgery are being more frequently indicated and have demonstrated significant efficacy^2^ ^,^ ^7^ ^,1^ ^9^.

For advanced cancers, the most successful treatments are combined chemotherapy with surgery^2^ ^,^ ^6^ ^,^ ^20^. However, there are few tools to restaging patients before the surgical procedure^10^. 

This study describe the pre-operative endoscopic findings of 29 patients with GC and GEJ cancer who were submitted to NAC and who achieved pCR after surgery. Methods for assessing tumor response and metastases after chemotherapy include endoscopic ultrasound, CT, and PET-CT, but these techniques show low accuracy and the possibility of over or under-staging^17^ ^,^ ^18^. 

The study included EGD performed by two independent senior endoscopists, after the neoadjuvant treatment. The exams were performed within 30 days of the end of NAC, and surgery was performed 4-8 weeks after the end of NAC.

The EGD was able to predict pCR in 65.5% of cases (G1). In addition, the time interval between the end of NAC and the performance of EGD2 was significantly shorter for the group in which EGD was unable to predict pCR (G2) and for each additional day there was a 12% increase in the probability of predicting pCR. 

The ideal time calculated to perform EGD was 8.5 days after ending NAC, at which point sensitivity reached 93%. The presence of active lesions in G2 may have been due to inflammatory responses that occur during normal healing of the mucosa. In these cases, EGD performed later may have revealed scarring compatible with that observed in G1.

## CONCLUSION

The eCR, determined by the presence of endoscopic scar, reddish or whitish, without active lesions after NAC, was consistent with the pCR. EGD after NAC showed accuracy in predicting eCR and sensitivity in 65.5% of the cases analyzed. The minimum time interval for performing EGD after the end of NAC was 8.5 days. Respecting this interval may increase the possibility of predicting pCR with endoscopic evaluation and supports optimal response prediction
